# SISTER OF TM3 activates *FRUITFULL1* to regulate inflorescence branching in tomato

**DOI:** 10.1038/s41438-021-00677-x

**Published:** 2021-12-01

**Authors:** Xiaotian Wang, Zhiqiang Liu, Shuai Sun, Jianxin Wu, Ren Li, Haijing Wang, Xia Cui

**Affiliations:** 1 Key Laboratory of Biology and Genetic Improvement of Horticultural Crops of the Ministry of Agriculture, Institute of Vegetables and Flowers, Chinese Academy of Agricultural Sciences, Beijing, 100081 China; 2 Sino-Dutch Joint Laboratory of Horticultural Genomics, Institute of Vegetables and Flowers, Chinese Academy of Agricultural Sciences, Beijing, 100081 China

**Keywords:** Gene regulation, Plant morphogenesis

## Abstract

Selection for favorable inflorescence architecture to improve yield is one of the crucial targets in crop breeding. Different tomato varieties require distinct inflorescence-branching structures to enhance productivity. While a few important genes for tomato inflorescence-branching development have been identified, the regulatory mechanism underlying inflorescence branching is still unclear. Here, we confirmed that SISTER OF TM3 (STM3), a homolog of *Arabidopsis* SOC1, is a major positive regulatory factor of tomato inflorescence architecture by map-based cloning. High expression levels of *STM3* underlie the highly inflorescence-branching phenotype in ST024. *STM3* is expressed in both vegetative and reproductive meristematic tissues and in leaf primordia and leaves, indicative of its function in flowering time and inflorescence-branching development. Transcriptome analysis shows that several floral development-related genes are affected by *STM3* mutation. Among them, *FRUITFULL1* (*FUL1*) is downregulated in *stm3cr* mutants, and its promoter is bound by STM3 by ChIP-qPCR analysis. EMSA and dual-luciferase reporter assays further confirmed that STM3 could directly bind the promoter region to activate *FUL1* expression. Mutation of *FUL1* could partially restore inflorescence-branching phenotypes caused by high *STM3* expression in ST024. Our findings provide insights into the molecular and genetic mechanisms underlying inflorescence development in tomato.

## Introduction

Higher plants exhibit various inflorescence architectures progressing in complexity from a solitary flower to complex structures that contain multiple branches and flowers. The architecture of inflorescences is one of the determinant traits for many crops, such as rice (*Oryza sativa*), maize (*Zea mays*), and tomato (*Solanum lycopersicum*)^1^. Favorable inflorescence branching is always a major breeding target for achieving desirable production by balancing the sink-source relationship. Distinguished from the raceme-type inflorescences of *Arabidopsis* and panicle-type inflorescences of rice, tomato has a cyme-type inflorescence lacking a main axis, which initiates from a sympodial inflorescence meristem (SIM). The SIM generates a new SIM before terminating in a floral meristem (FM) and reiteration of this pattern produces tomato inflorescences and determination of branching^1–4^.

A series of regulatory genes that have received much attention makes major contributions to inflorescence architecture in tomato by changing the inflorescence-branching pattern. A larger vegetative shoot apical meristem (SAM) often produces more branching inflorescences. The WUSCHEL-CLAVATA (WUS-CLV) feedback regulatory loop is conserved maintaining the balance among SAM activities and controlling meristem size in *Arabidopsis*, rice, tomato, and other plants^5–11^. Mutations in the CLV pathway genes, *SlCLV3*, *FASCIATED AND BRANCHED* (*FAB*), and *FASCIATED INFLORESCENCE* (*FIN*), cause meristems to enlarge, leading to an increase in inflorescence branching in tomato^12,13^.

During phase transitions, flowering time genes are also important players in regulating inflorescence architecture in tomato. The florigen mutant, *single-flower truss* (*sft*), is late-flowering and disrupts normal tomato sympodial growth, which reverses the inflorescence toward vegetative functioning after the initiation of one or a few flowers depending on the growing conditions^14–16^. The *jointless* (*j*) mutant produces indeterminate inflorescences that revert to vegetative growth after the production of two or three flowers^16,17^. Moreover, J and SFT cooperatively regulate the architecture of inflorescences preventing early changes in inflorescence meristem (IM) identity once inflorescence morphogenesis is initiated^18^. In addition, *FALSIFLORA* (*FA*), the tomato ortholog *LEAFY*, controls flowering time and floral meristem identity. The *FA* mutation results in the conversion of flowers into secondary shoots and produces a highly branched inflorescence^19,20^. In addition to these promotion-flowering genes, *TERMINATING FLOWER* (*TMF*) encodes an ALOG family protein and affects inflorescence organization in tomato. The *tmf* mutants flower early and convert multiflowered inflorescence into a solitary flower^21^. TMF and SlBOPs act synergistically to prevent precocious flowering and promote inflorescence complexity. All inflorescences on the *slbop1/2/3* triple mutant develop only one or two flowers^22^.

In addition, floral meristem identity genes have a profound influence on tomato inflorescence architecture. Loss of *ANANTHA* (*AN*) and *COMPOUND INFLORESCENCE* (*S*) delay the progression of an IM to the floral meristem (FM), resulting in additional branching^3,23^. JOINTLESS2 (J2), ENHANCER-OF-JOINTLESS2 (EJ2) and LONG INFLORESCENCE (LIN), three SEPALLATA 4 (SEP4) proteins, have redundant roles in inflorescence branching and cause a quantitative range of inflorescence branching with combinations of homozygous and heterozygous *J2* and *EJ2* mutations in tomato^24–26^. However, reconfiguration of inflorescence branching for breeding higher yield varieties in tomato remains challenging due to the limited known loci or genes and the largely unknown underlying mechanisms.

SUPPRESSOR OF OVEREXPRESSION OF CONSTANS 1 (SOC1) was first identified as an important flowering time integrator that regulates the floral transition in *Arabidopsis*^27–29^. In addition, SOC1 and three MADS-box transcription factors, SHORT VEGETATIVE PHASE (SVP), AGAMOUS-LIKE 24 (AGL24), and SEP4, act redundantly and directly suppress *TERMINAL FLOWER 1* (*TFL1*) to control *Arabidopsis* inflorescence architecture^30^. *STM3*, a homolog of SOC1 in tomato, also regulates inflorescence branching^26^. However, the mechanism by which STM3 positively regulates inflorescence branching in tomato is still unknown. In our study, we confirmed that STM3 is a major positive factor controlling inflorescence branching in tomato. Mutation of *STM3* reduced the number of inflorescence branches, and overexpression of *STM3* produced compound inflorescences. STM3 directly binds the promoter of *FRUITFULL1* (*FUL1*) and activates its expression in vitro and in vivo. Mutation of *FUL1* could partially restore inflorescence-branching phenotypes caused by high *STM3* expression. Our findings provide insights into the molecular and genetic mechanisms underlying inflorescence development in tomato.

## Results

### *qMIB1* controls inflorescence branching in tomato

**Fig. 1:**
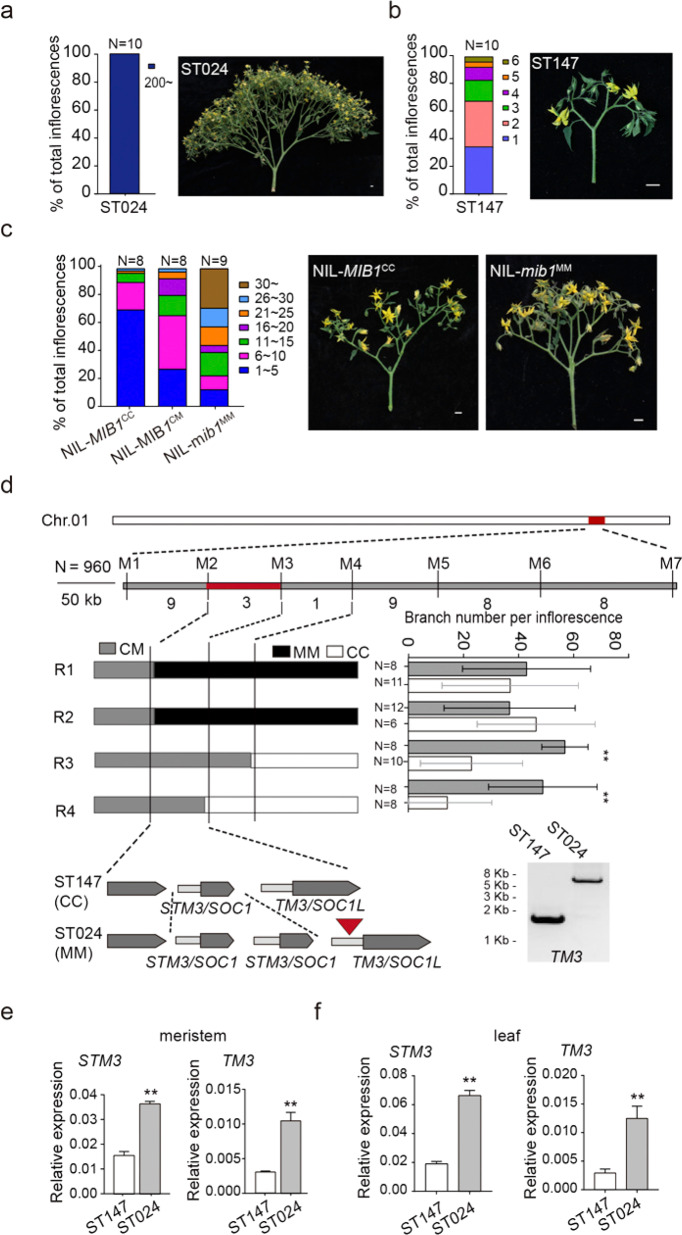
Identification and Characterization of *qMIB1*. **a**, **b** Quantification of inflorescence branching and representative images from the two parent lines: ST024 (**a**) and ST147 (**b**). **c** The phenotype of near-isogenic lines. NIL-*MIB1*^*CC*^ and NIL-*mib1*^*MM*^ represent the ST147 and ST024 genotypes, respectively, and CM is a heterozygous genotype. **d** Fine mapping of *qMIB1*. Top panel, positional cloning narrowed *qMIB1* to the DNA segment between markers M2 and M3. The numbers below the bars indicate the number of recombinants. Middle panel, high-resolution mapping of *qMIB1* (left), and the progeny test of four recombinants (right). The black, gray, and white boxes indicate chromosome regions with the MM, CM, and CC genotypes, respectively. N, plant number. Bottom panel, genomic diagram showing the candidate ORFs. The gel picture showed a large insertion in the *TM3* promoter of ST024. **e**, **f** Quantification of the relative expression of *STM3* and *TM3* in reproductive meristematic tissues and leaves of 21-d-old seedlings between ST147 and ST024. *UBIQUITIN3* was used as the internal control. Data were compared by two-tailed Student’s *t*-test, **P < 0.01, error bar, SD

To identify the loci regulating tomato inflorescence branching, we found a line, ST024, which has extraordinary inflorescence branching with long sepals and jointless pedicels that are similar to the *j2* and *ej2* mutants^24,31^, from a set of 201 stable recombinant inbred lines (RILs) generated from a cross of *Solanum Lycopersicum var. cerasiforme* LA1310 (CC) and *S. Lycopersicum* Moneymaker (MM)^32^. The parental CC was generally a simple inflorescence, and less than 20% of inflorescences had two branches. Compared with CC, MM showed complex inflorescence branching, and more than 60% of inflorescences had more than two branches (Fig. 1a; Supplementary Fig. S1a−c). The genome sequencing data of ST024 and genotyping revealed a 5 bp deletion in the 4^th^ exon of *J2* leading to early termination of its translation, which was named *j2*^*del*^, and a 564 bp insertion in the 5^th^ intron of *EJ2*, similar to the previously reported weak *ej2*^*W*^ mutant^24^ that resulted in a jointless pedicel and long sepal of ST024 (Supplementary Fig. S1d−f). Based on RIL population sequencing data, we screened ten lines with the same *j2*^*del*^*ej2*^*W*^ genotypes^32^, unexpectedly, these RILs with the same *j2*^*del*^*ej2*^*W*^ genotypes showed different inflorescence architectures (Supplementary Fig. S1g), suggesting that additional variants influence inflorescence diversity in RILs.

To explore the genetic basis of the branch discrepancy among these RILs, we crossed ST024 with ST147, one of the RILs with the *j2*^*del*^*ej2*^*W*^ genotype, and a weakly branched inflorescence (Fig. 1b). We sequenced pools of DNA from two subcategorized groups of the F_2_ population that exhibited extreme inflorescence architecture. One locus, *qMIB1* (QTL for *MULTIPLE INFLORESCENCE BRANCH 1*), on chromosome 1 was identified (Supplementary Fig. S2a). To further investigate the contribution of *qMIB1*, we generated NILs by crossing ST147 and ST024 and selected offspring heterozygous at the *qMIB1* locus. After six generations of selfing, F_7_ with a 191 kb heterozygous region around *qMIB1* was obtained. The progenies with different genotypes in this region were defined as near-isogenic lines (NILs) by marker screening. We compared inflorescence-branching number among NIL-*mib1*^MM^, NIL-*MIB1*^CC^, and NIL-*MIB1*^CM^ and found that NIL-*mib1*^MM^ plants had significantly more branches than NIL-*MIB1*^CC^ plants, and the heterozygous NIL-*MIB1*^CM^ line showed an intermediate extent of inflorescence branching that is consistent with F_1_ hybrids (Fig. 1c; Supplementary Fig. S2b, c), indicating that *qMIB1* is partially dominant.

### Fine mapping of *qMIB1*

Using 960 individuals of the F_2_ population, *qMIB1* was finally narrowed to a 76.12 kb region containing three annotated genes: *Solyc01g091950* and two neighboring MADS-box genes, *Solyc01g092950* (*STM3*) and *Solyc01g093965* (*TM3*) (Fig. 1d; Supplementary Fig. S3a). We obtained the sequence of the *STM3* transcript (Supplementary Fig. S3b, c) by RACE and found no variations in the coding regions of these two genes between ST024 and ST147. The sequence data for these lines and our PCR results revealed an ~20 kb repeat sequence at this region in the ST024 genome but not in ST147, which is consistent with a recent study that demonstrated that structural variation in the tomato genome resulted in an additional copy of *STM3*^26^. In addition, we found one gap in the promoter of *TM3* according to the SL2.50 reference genome. Using two specific primers on the two sides of the gap, an ~7 kb DNA fragment was amplified in ST024/MM, but only a 1.7 kb DNA fragment was obtained in ST147/CC (Fig. 1d and Supplementary Fig. S4a), suggesting that the gap was caused by an ~5.3 kb DNA fragment insertion. To test whether the repeat and insertion affect *STM3* or *TM3* expression, we detected the expression levels of *STM3* and *TM3* in reproductive meristematic tissues and leaves of 21-d-old seedlings by quantitative real-time PCR (qRT-PCR) and found that the expression levels of both *STM3* and *TM3* were higher in ST024 than in ST147. Higher expression levels were also detected in NIL-*mib1*^MM^ than in NIL-*MIB1*^CC^ (Fig. 1e, f; Supplementary Fig. S4b, c). These results suggested that high expression of *STM3* and *TM3* caused by these sequence variations may underlie the more inflorescence-branching phenotype in ST024.

### *STM3 and TM3* redundantly regulate inflorescence branching in tomato

**Fig. 2:**
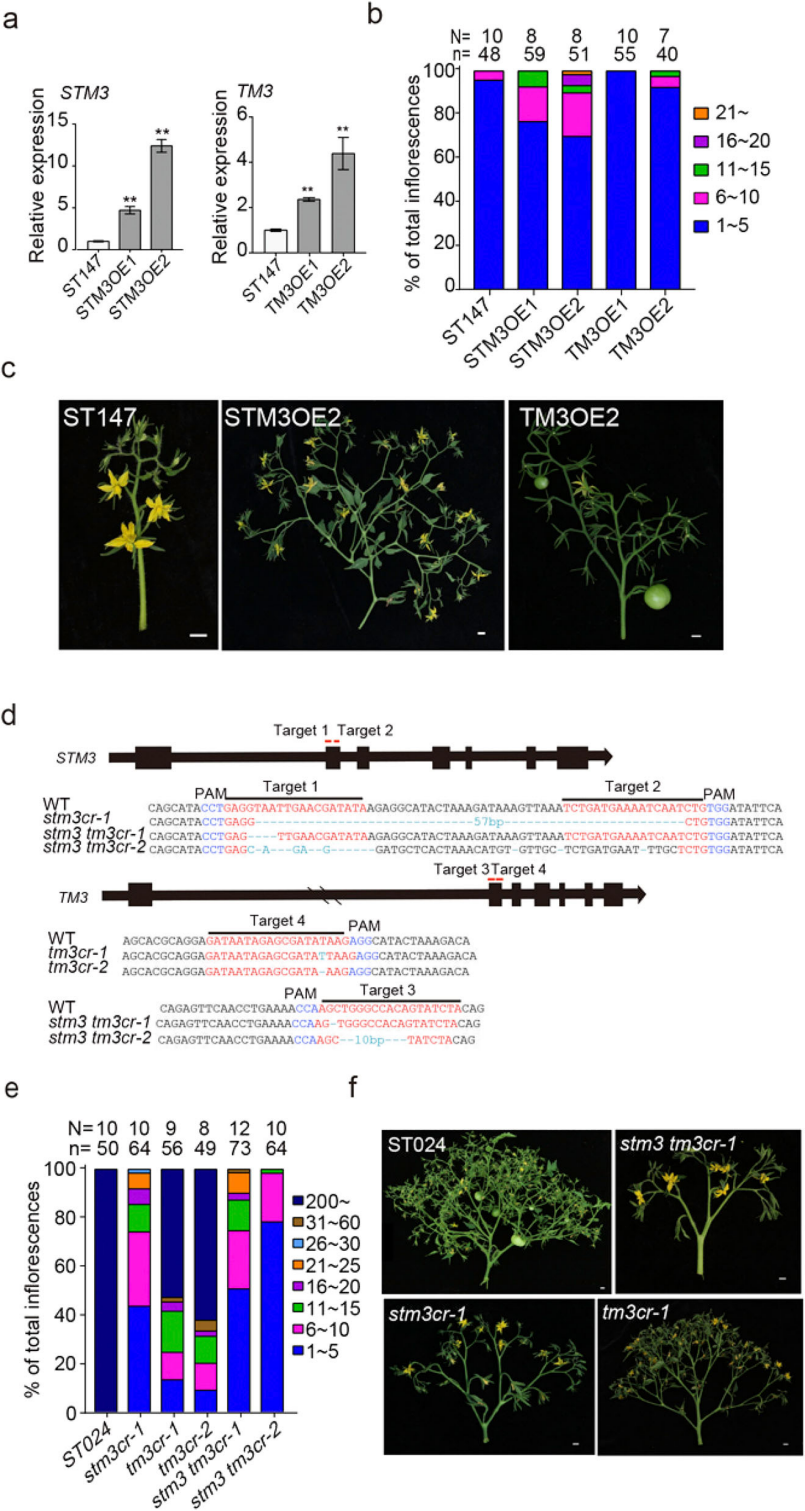
STM3 and TM3 function redundantly to control inflorescence branching in tomato. **a** Expression levels of *STM3* and *TM3* in overexpressed transgenic plants. **b** Quantification of inflorescence branching in ST147 and overexpressed transgenic plants. **c** Representative inflorescence images of ST147 and overexpressed transgenic plants. **d** *STM3* and *TM3* mutations generated by CRISPR/Cas9. Red words mark the position of targets. Blue words indicate the protospacer-adjacent motif (PAM). Green words mark the mutation sequences of one *stm3cr-1* mutant, two *tm3cr* mutants, and two *stm3 tm3cr* mutants in ST024. **e** Quantification of inflorescence branching in ST024, the single and double mutants. **f** Representative inflorescence images of all mutants. N, plant number, n, inflorescence number, scale bars = 1 cm

To verify that the higher expression of *STM3* and *TM3* led to more inflorescence branching in ST024, we transformed ST147 with vectors carrying the full-length CDS of *TM3* and S*TM3* fused with YFP-HA or GFP-FLAG tags, respectively, and driven by the 35S promoter. Two independent overexpressed transgenic lines for each construct were obtained (Fig. 2a). As expected, the STM3OE transgenic plants exhibited higher branching inflorescence than ST147 but were still far less than ST024, whereas the TM3OE lines had no obvious differences from ST147 (Fig. 2b, c). In addition, we also generated single null mutants, *stm3cr*-1, *tm3cr-1,* and *tm3cr-2*, and the null double mutants *stm3 tm3cr*-1 and *stm3 tm3cr*-2 using CRISPR/Cas9 in the ST024 background (Fig. 2d). Intriguingly, reduced inflorescence branching was observed in all single and double mutants; yet *stm3cr* single and *stm3 tm3cr* double mutants had strongly suppressed branching, *tm3*cr mutant had weakly suppressed branching (Fig. 2e, f). These results suggested that *STM3* and *TM3* may have at least partially redundant functions in controlling inflorescence-branching development.

It should also be noted that only the *stm3 tm3cr* and *stm3cr* plants photocopied a late-flowering phenotype, which is consistent with a recent study that demonstrated a delayed flowering phenotype in lines with the *sb1*^*CR-1*^ and *sb1*^*CR-*del^ mutants^26^ (Supplementary Fig. S5), but there was no obvious change in *tm3cr* compared with ST024.

### *STM3* expressed in tomato different meristems and tissues

**Fig. 3:**
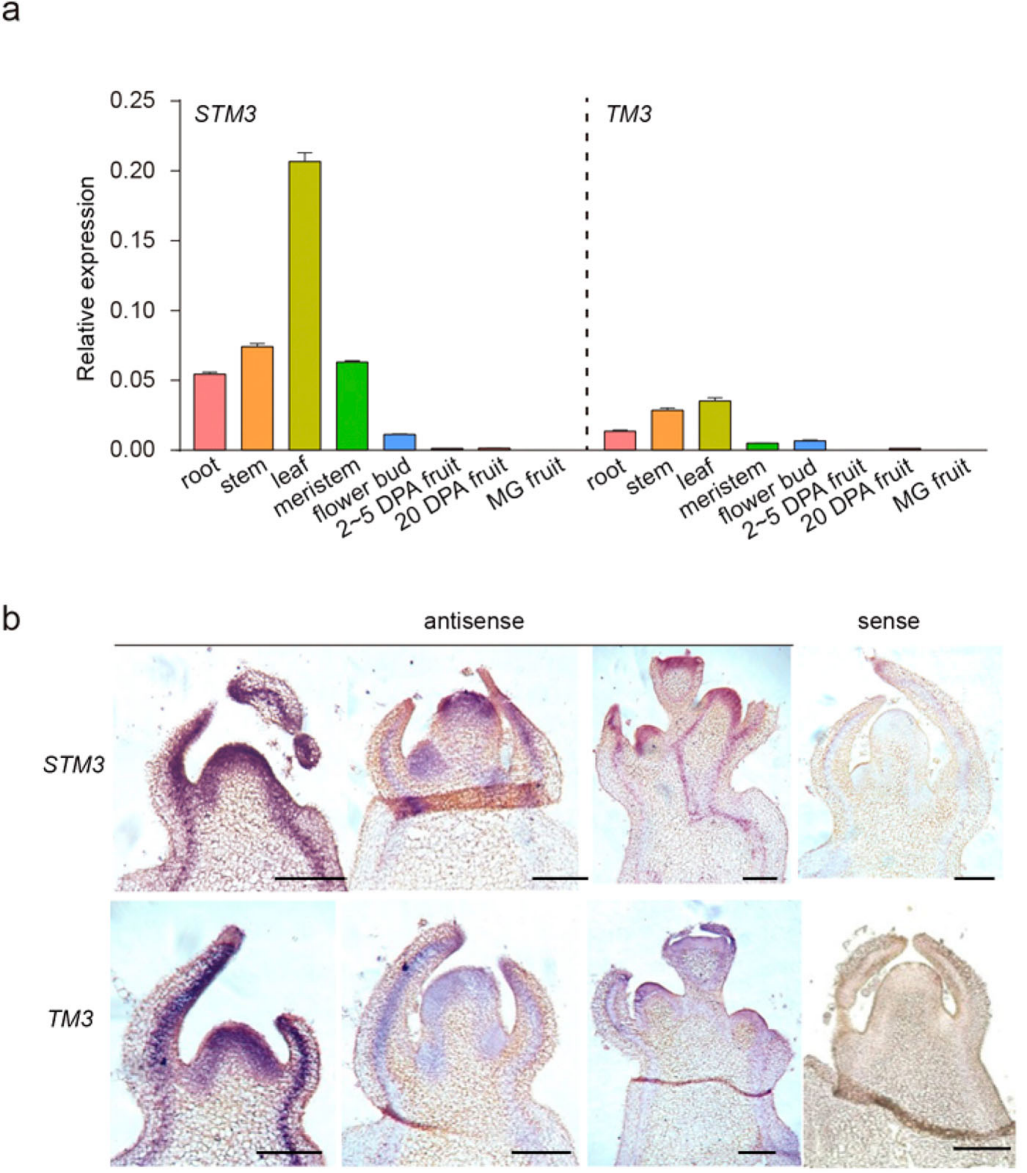
STM3 is expressed in vegetable and reproductive meristems. **a** qRT-PCR for *STM3* and *TM3* expression in root, stem, leaf, meristem, flower, and fruit. The root, stem, leaf, and meristem are from 30-d-old seedlings. DPA, days post anthesis. MG mature green. *UBIQUTIN3* was used as the internal control. Error bar, SD. **b** In situ mRNA hybridization of *STM3* and *TM3* mRNA transcripts in vegetative and reproductive meristems of MM. Scale bars = 100 μm

To better understand the individual and combined roles of tomato *STM3* and *TM3*, we first analyzed their expression patterns in different organs. Both genes were expressed in roots, flowers, and meristems, with especially strong expression in leaves (Fig. 3a), an expression profile consistent with *SOC1* from *Arabidopsis*^33^. Moreover, in situ hybridization analysis of longitudinal sections of various meristems showed that *STM3* and *TM3* exhibit similar expression profiles, with strong accumulation at apices of meristems, and both accumulated in leaf primordia and vascular bundles (Fig. 3b), supporting their roles in inflorescence development. Without exception, the expression level of *STM3* was higher than that of *TM3* in all tissues, which is in line with their functions in inflorescence architecture and flowering.

### STM3 physically interacts with TM3

**Fig. 4:**
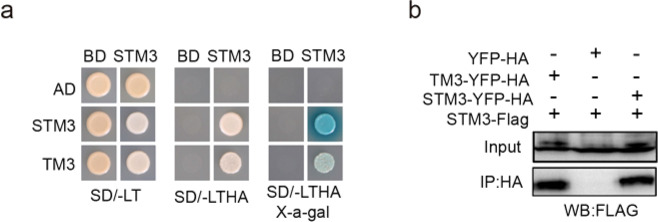
STM3 interacts with TM3 and itself. **a** Interaction assays for the STM3 and TM3 proteins in yeast cells. Yeast cells grown on a selective medium (-LTHA) showed a positive interaction. -LT, without Leu and Trp. -LTHA, without Leu, Trp, His, and Ade. **b** Co-immunoprecipitation assays to examine interactions between STM3 and TM3 in *N. benthamiana* leaves. Immunoblots of the total protein extracts (Input) and the IP product were performed using an anti-FLAG antibody (α-FLAG)

MADS-box proteins often constitute multimeric complexes to direct a variety of developmental processes^34^. Therefore, we tested the interaction between STM3 and TM3 by yeast two-hybrid assay. Our results indicated that STM3 was able to interact with TM3 and itself (Fig. 4a). Further co-immunoprecipitation (Co-IP) assays using tobacco (*Nicotiana benthamiana*) leaves, in which STM3-FLAG was transiently expressed together with YFP-HA, STM3-YFP-HA, and TM3-YFP-HA, confirmed that STM3 could be immunoprecipitated by STM3-YFP-HA and TM3-YFP-HA fusion proteins but not by the YFP-HA control (Fig. 4b). These results further indicated that STM3 and TM3 control inflorescence architecture together.

### STM3 regulates multiple biological processes during inflorescence development

**Fig. 5:**
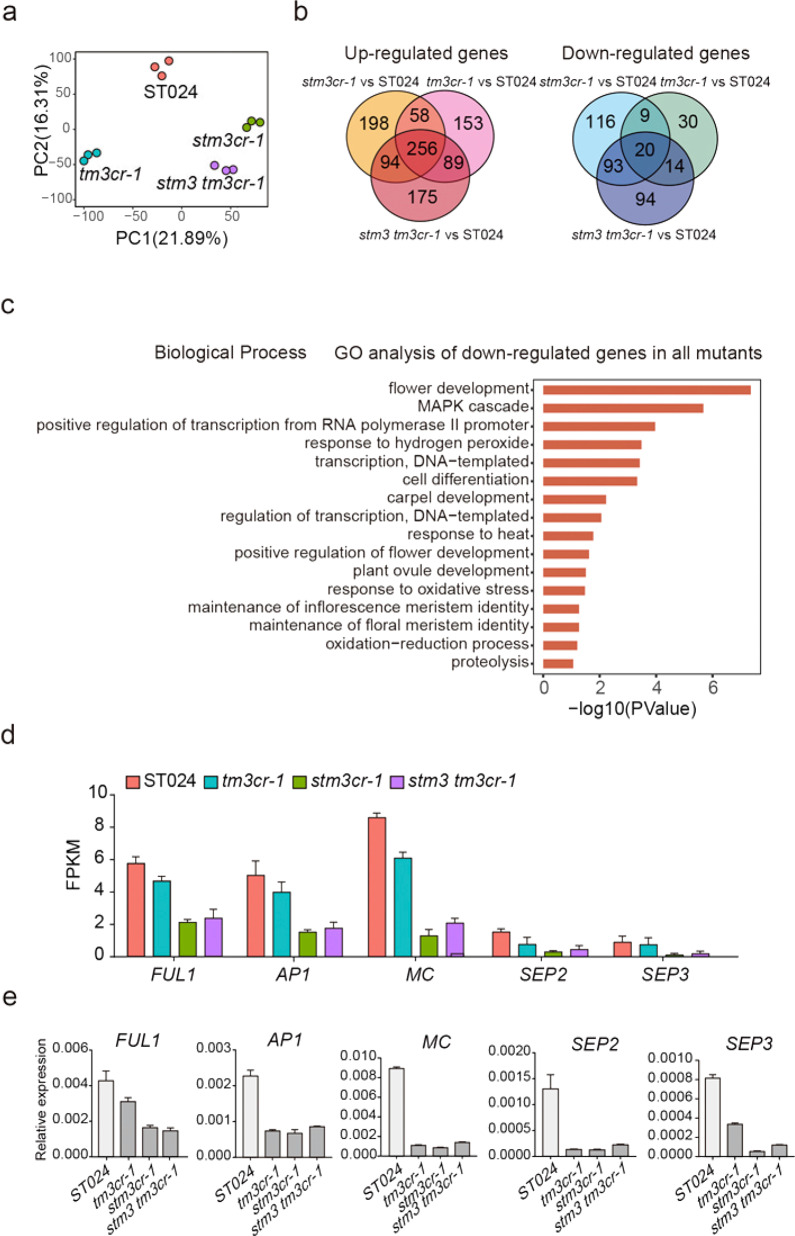
STM3 regulates multiple biological processes during inflorescence development. **a** Principal component analysis (PCA) of RNA-seq data. **b** Venn diagram showing differentially expressed genes among ST024, *tm3cr-1*, *stm3cr-1,* and *stm3 tm3cr*-1. **c** GO enrichment analysis of downregulated genes in *tm3cr-1*, *stm3cr-1,* and *stm3 tm3cr-1* compared with ST024. **d** FPKM values of DEGs for floral meristem identity between ST024 and *tm3cr-1* and *stm3cr-1* and *stm3 tm3cr-1*. **e** qRT-PCR verification of DEGs identified by RNA-seq analysis. Data were compared by two-tailed Student’s *t*-test, **P < 0.01, error bar, SD.

To investigate the molecular mechanism of STM3 in the regulation of inflorescence development, we collected reproductive meristematic tissues of ST024, *tm3cr-1*, *stm3cr-1,* and *stm3 tm3cr-1* to perform RNA-seq analysis with three biological replicates. Here, approximately 30 million uniquely mapped reads were generated for each sample (Supplementary Table S1). The principal component analysis (PCA) results showed that the sequencing data were highly reproducible (Fig. 5a). From the PCA, the *stm3cr-1* and *stm3 tm3cr-1* mutants were located closely, which is in a sense consistent with the inflorescence-branching phenotype. In total, compared with ST024, 629, 844, and 835 differentially expressed genes (DEGs) were identified in *tm3cr-1*, *stm3cr-1,* and *stm3 tm3cr-1*, respectively (Fig. 5b). We performed Gene Ontology (GO) term enrichment analysis using DAVID to gain insight into the functions of these DEGs between ST024 and the single and double mutants (Supplementary Table S2). In particular, flower development-related, cell differentiation, transcription-related, and maintenance of inflorescence and floral meristem identity terms were enriched in the downregulated genes (Fig. 5c; Supplementary Figs. S6–8). In contrast to the downregulated genes, the development-related process was rarely enriched in the upregulated genes in the single and double mutants, but most genes related to biotic or abiotic response pathways were enriched (Supplementary Figs. S6–8). We further analyzed these 136 downregulated DEGs, which were repressed in any two or more mutants. Among these genes, *FUL1* (*Solyc06g069430*), *APETALA1*/*AP1* (*Solyc02g089210* *MACROCALYX/MC* (*Solyc05g056620*), *SEPALLATA2/SEP2* (*Solyc02g089200*), and *SEPALLATA3/SEP3* (*Solyc05g015750*), whose *Arabidopsis* homologs are related to floral meristem establishment, showed reduced expression levels in the single and double mutants^35^ (Fig. 5d). Consistent with the RNA-seq results, our qRT-PCR assays confirmed the differential expression of these genes (Fig. 5e), suggesting that STM3 may regulate inflorescence development through interactions with other developmental regulators.

### STM3 targets *FUL1* to activate its expression

**Fig. 6:**
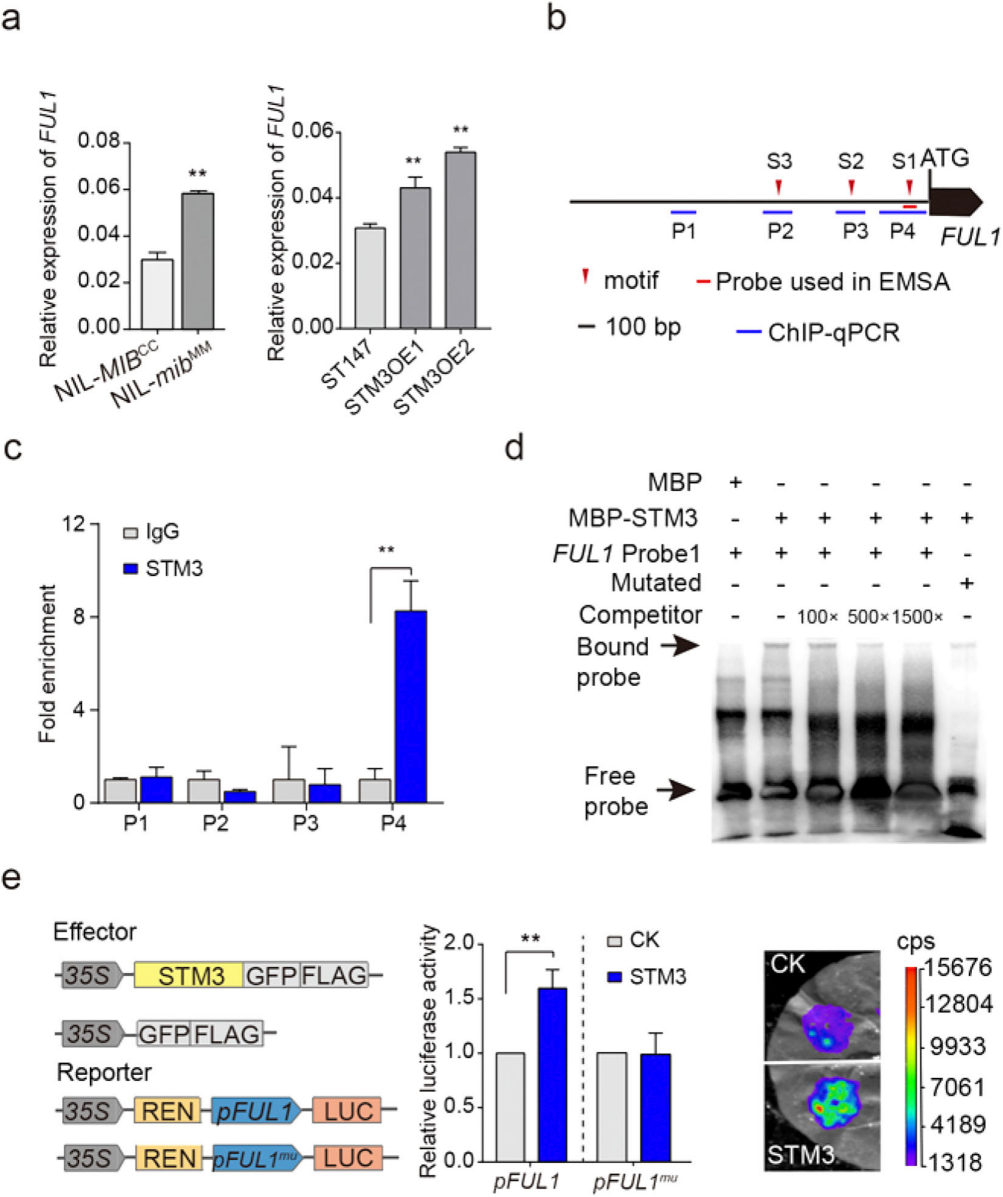
STM3 directly activates the expression of *FUL1*. **a** Relative expression of *FUL1* in reproductive meristematic tissues of NIL-*MIB1*^CC^, NIL-*mib1*^MM^*,* and two *STM3OE* lines. **b** Schematic diagram of the promoters of *FUL1*. Red triangles indicate the SOC1-binding motif. Blue lines indicate primers used for ChIP-qPCR assay, and short red lines indicate DNA probes used for EMSA. The translational start site (ATG) is shown at position +1. **c** ChIP-qPCR assays showing the enrichment of STM3 at the promoter of *FUL1*. The immunoprecipitated chromatin was analyzed by qPCR using gene-specific primers as indicated in (**b**), and IgG was used as the negative control. The intergenic region around *ACTIN* was used as an internal control. Error bars represent the SD of three biological replicates. Data were compared by two-tailed Student’s *t*-test, **P < 0.01, error bar, SD. **d** EMSA showing that STM3 interacts with the probe containing the CArG box on the *FUL1* promoter. **e** Dual-luciferase reporter assay showing that STM3 activates *FUL1* expression in tobacco leaves. Fold changes of relative luciferase activity are shown; mu, mutated. Data were compared by two-tailed paired Student’s *t*-test, **P < 0.01, error bar, SEM.

*FUL1* is the tomato homolog of *Arabidopsis FUL*, which has been demonstrated to regulate inflorescence architecture in *Arabidopsis*^36–39^. In tomato, the *FUL1* transcript accumulates gradually from the vegetable to reproductive stages and reaches the highest level at the floral meristem^23^, implying that it functions in inflorescence development. In contrast to its reduced expression in the *stm3cr-1* and *stm3 tm3cr-1* mutants, the expression level of *FUL1* was enhanced in the NIL-*mib1*^MM^ plants and STM3OE lines, in which *STM3* was highly expressed, compared with NIL-*MIB1*^CC^ and ST147 plants (Fig. 6a). Therefore, *FUL1* should be one of the downstream genes of STM3.

As transcription factors, MADS-box proteins could directly bind to gene promoters to regulate their transcription. Similar to other MADS-box proteins, *Arabidopsis* SOC1 recognizes the CArG box to directly bind its targets^28,29^. To explore whether *FUL1* is bound by STM3, we analyzed the promoter sequence of tomato *FUL1*, and three putative SOC1-binding motifs (S1, S2, and S3) were identified on the *FUL1* promoter by JASPAR^40^ (Fig. 6b). We further performed chromatin immunoprecipitation (ChIP) using an HA antibody against the HA-tagged STM3 protein in *35S*::*STM3-YFP-HA* transgenic plants. Obvious enrichment was observed in P4 around S1 but not in the other regions of the *FUL1* promoter by ChIP-qPCR analysis (Fig. 6c), indicating that STM3 directly targets *FUL1* in tomato. To determine whether STM3 can directly bind the motif, we performed electrophoretic mobility shift assays (EMSAs) using a 49 bp DNA fragment of the *FUL1* promoter containing S1 and the recombinant protein of STM3 with an MBP tag at the N-terminus (MBP-STM3). We found that MBP-STM3 but not MBP bound the *FUL1* probe. In addition, the excess unlabeled oligonucleotide probe can compete with the labeled probe, and with the increased content of the competed unlabeled oligonucleotide probe, fewer labeled probes are bound by STM3. Moreover, when the CArG box motif of the probe was mutated, the binding of STM3 was obviously reduced (Fig. 6d). All results showed that STM3 could directly bind the *FUL1* promoter by recognizing the CArG box.

To test how STM3 regulates *FUL1* expression, we performed a transient expression assay by coexpressing STM3 and the *FUL1* promoter in tobacco. Significantly increased luminescence intensity was observed upon coexpression of the STM3 and *FUL1* promoters in tobacco leaves compared with the control upon coexpression of GFP-FLAG and the *FUL1* promoter. However, no significant differences were observed when coexpressed STM3 and mutated *FUL1* promoter (Fig. 6e). In line with the decreased expression level of *FUL1* in the *stm3cr-1* mutants, these results further confirmed that STM3 activates *FUL1* transcription in vivo by binding the CArG box.

### *FUL1*, as a target of STM3, regulates inflorescence branching in tomato

**Fig. 7:**
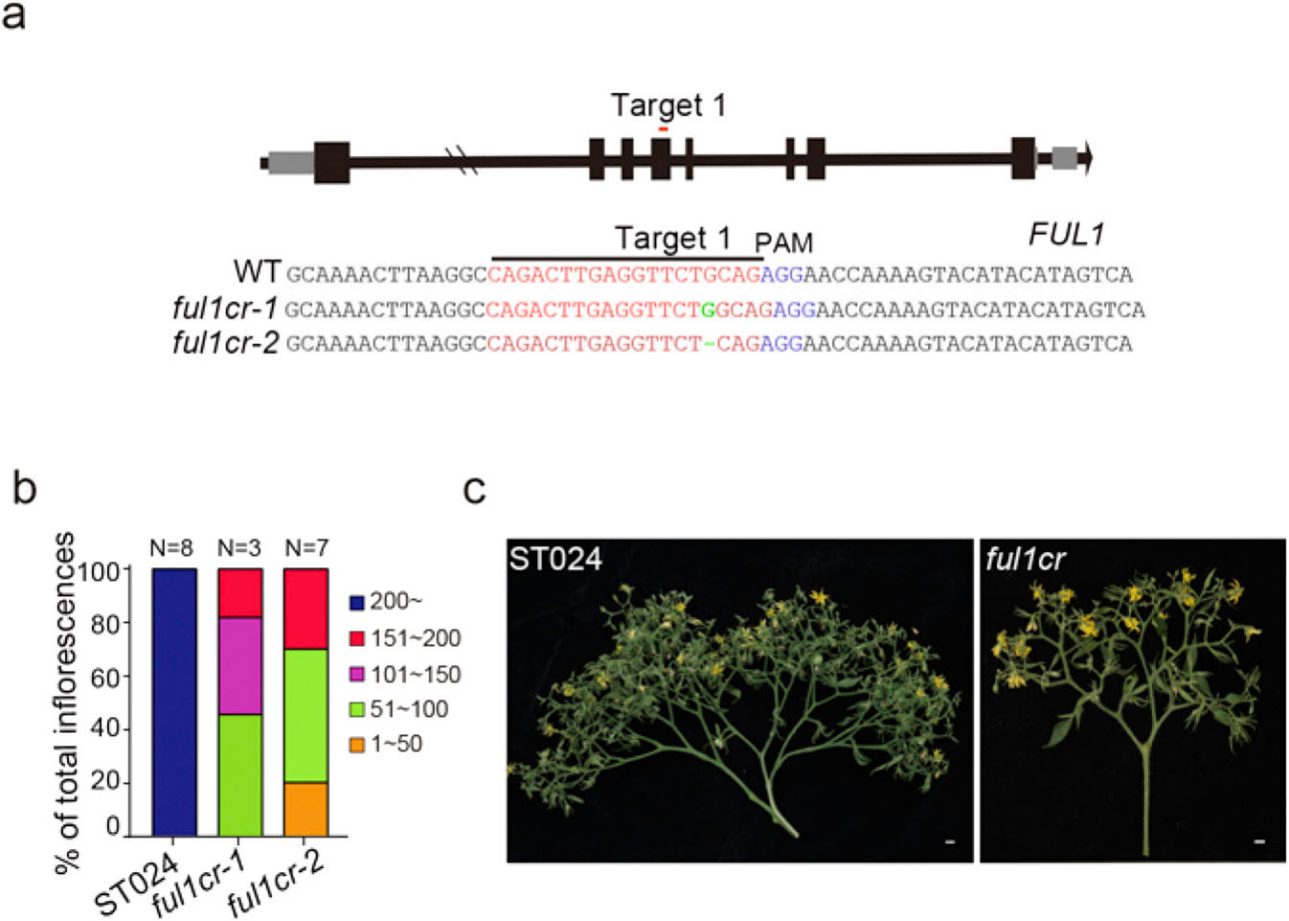
FUL1 regulates inflorescence-branching development in tomato. **a** *FUL1* mutations generated by CRISPR/Cas9. Red letters mark the position of targets. Blue letters indicate the protospacer-adjacent motif (PAM). Green letters mark the mutation sequences of *ful1* mutants in ST024. **b** Quantification of inflorescence branching in ST024 and *ful1cr* mutants. *N*, plant number. **c** Representative inflorescence images of ST024 and *ful1cr* mutants. Scale bars = 1 cm

To further characterize the role of FUL in tomato inflorescence development, we used CRISPR/Cas9 to engineer *FUL1* in ST024 plants. Two null alleles, *ful1cr-1* and *ful1cr-2*, with 1 bp insertion and 1 bp deletion, were obtained (Fig. 7a). Compared with ST024 plants, the *ful1cr-1* and *ful1cr-2* engineered plants exhibited reduced inflorescence branching. However, their inflorescence architectures were still more complicated than those of ST147 and *stm3cr-1* plants in the ST024 background, indicating that knocking out *FUL1* only partially suppressed inflorescence branching (Fig. 7b, c). These results suggested that STM3 regulates inflorescence development to a certain extent through its target, *FUL1*.

## Discussion

Similar to other plants, tomato inflorescence architecture is governed by inflorescence meristems and their derived meristems between maintenance of indeterminacy and commitment to floral fate^4^. Our studies reveal that TM3 and STM3, two homologs of Arabidopsis SOC1, redundantly regulate tomato inflorescence branching, which is similar to a recent study reporting that natural variation in STM3 causes its higher expression, leading to higher inflorescence branching in tomato^26^. Although TM3 has a redundant function with STM3 in inflorescence development, *TM3* overexpression alone could not induce more inflorescence branching in ST147, and the inflorescence architecture of the *tm3cr* mutant was weaker than that of the *stm3cr* mutant, suggesting that *STM3* is a major gene controlling inflorescence branching in tomato. Consistent with their functions, the expression level of *STM3* was obviously higher than that of *TM3* in all tissues, although they showed similar expression patterns. Moreover, our results also indicated that SOC1s promote inflorescence branching in a dose-dependent manner in tomato. The moderate branching of F_1_ plants and the various branched phenotypes of the *STM3* overexpression transgenic plants were positively related to the expression levels of *STM3*.

SOC1 is an important activator integrating floral inductive signals from multiple pathways to promote flowering in many plants^27,41–44^. In contrast to only one SOC1 in Arabidopsis that controls flowering^45^, three homologs of *SOC1* are in the tomato genome (Supplementary Fig. S2a). Our results indicate that STM3 has a similar function in flowering time and that its null mutants flowered later in tomato (Supplementary Fig. S5). However, the flowering time of the *tm3cr* mutant was almost unaffected, suggesting that the functions of STM3 and TM3 were partly segregated in tomato. In *Arabidopsis*, SOC1 also functions in inflorescence architecture and acts redundantly with three other MADS-box transcription factors, SEP4, AP1, and SVP, to repress inflorescence branching. In contrast to the *stm3cr* and *tm3cr* mutants showing reduced inflorescence branching, the quadruple mutant shows a massive inflorescence-branching phenotype^30^, indicating that STM3 and its *Arabidopsis* homolog play reverse functions in regulating inflorescence-branching development. Therefore, the diverse functions of STM3 imply distinct mechanisms underlying tomato and *Arabidopsis* inflorescence development.

As a MADS-box protein, SOC1 can directly target downstream genes by binding to the CArG box motif. Hundreds of SOC1 targets have been identified in *Arabidopsis* by ChIP-seq, including several genes that regulate flowering time and meristem maintenance^28,29^. Similarly, we found that STM3 could regulate the expression levels of flower development- and meristem maintenance-related genes according to our RNA-seq data. In addition, it is worth noting that *FUL1* is also repressed in the *stm3cr-1* reproductive meristems, which was previously reported to be expressed during ripening and interact with RIN to mediate ethylene-independent aspects of fruit ripening in tomato^46^. We found that *FUL1* is directly bound by STM3 through one of the CArG boxes on its promoter region in tomato, further supporting that *FUL1* is regulated by STM3 in tomato meristems. In accordance with the role of FUL1 in inflorescence branching, *FUL1* is also expressed in vegetative and reproductive meristems^23^, and the *ful1cr* mutants could partially rescue the massive inflorescence branching of ST024. However, the inflorescence architectures of *ful1cr* mutants are still more complicated than those of *stm3cr* mutants, suggesting that *FUL1* is one of the downstream targets of STM3 in tomato inflorescence development. In the future, the identification of more direct targets of STM3 will help us to understand the molecular mechanisms and regulatory network of inflorescence development in tomato.

## Materials and methods

### Plant materials and growth conditions

RILs derived from *S. Lycopersicum cv*. Moneymaker (MM) and *S. Lycopersicum var. cerasiforme* LA1310 (CC) were used as materials for the assessment of inflorescence branches as we described previously^32^. The NILs were generated by crossing ST147 and ST024. Then, heterozygous offspring at the q*MIB1* locus were selected for selfing. After six generations, we used markers to select plants homozygous for *MIB1*^CC^ or *mib1*^MM^ and to determine the introgressed region (~191 kb) around the q*MIB1* locus. Seedlings of RILs, NILs, and transgenic plants were grown in a commercial nursery for 30–40 days and then transplanted to fields. For inflorescence-branching assessment, all tomato plants were grown in a greenhouse at the Shunyi experimental station in Beijing, China. At least six individual plants of each accession were used for inflorescence-branching examination. From each plant, 5−6 inflorescences were evaluated. The % of total inflorescences represents the proportion of the number of inflorescences within a certain range to a total number of inflorescences.

### Fine mapping of *qMIB1*

An F_2_ population derived from the cross between two RILs, ST147 and ST024, with the *j2*^*del*^*ej2*^*W*^ genotype was used for mapping. We selected 24 plants with extremely compound inflorescences (mean number of inflorescence branches >200) and 31 plants with simple inflorescences (mean number of inflorescence branches <1.5) for bulked segregant analysis (BSA). Equal amounts of tissue from each plant were pooled for DNA extraction using standard protocols. The genomic DNA was sheared using a Diagenode Bioruptor Plus instrument to obtain ~300 bp fragments. Libraries were prepared using the NEXTflex^TM^ Rapid DNA-Seq Kit for Illumina (NOVA-5144-08) according to the manufacturer’s protocol. Genomic DNA reads were trimmed by quality using Trimmomatic^47^^,^ and paired reads were mapped to the reference tomato genome (SL2.50) using BWA-MEM^48^. SNP calling was performed as described previously^49^. BSA was performed with modification^50^. SNPs between two parental genomes were identified for further analysis when the base quality value was ≥20 and the SNP quality value was ≥20. On the basis of these criteria and the number of SNPs with a read depth ≥5, an SNP index was calculated for both bulk samples expressing the proportion of reads harboring SNPs that were identical to those in the parent (ST147). The ∆SNP index was obtained by subtracting the SNP index for the simple inflorescence bulk sample from that for the compound inflorescence bulk sample. An average SNP index for the compound and simple inflorescence bulk samples was calculated using a 1,000 kb sliding window with a step size of 10 kb. For fine mapping *qMIB1*, 38 recombinant plants were selected according to the region determined by BSA-seq from 960 F_2_ seedlings and self-pollinated in the greenhouse. The progeny of ten recombinant plants was selected, and their inflorescence-branching number was observed.

### RNA extraction and gene expression analysis

RNA was isolated using TRIzol Universal reagent (Tiangen, DP424). DNA contamination was removed using the TURBO DNA-free Kit (Ambion, AM1907). Reverse transcription was performed with 5×All-In-One MasterMix with AccuRT Genomic DNA Removal Kit (abm, G492) using 2 μg of total RNA. Quantitative PCR (qPCR) was performed with EvaGreen 2×qPCR MasterMix-No Dye (Abm, MasterMix-S) on a Bio-Rad CFX-96 Real-Time PCR instrument using the following program: 3 min at 95 °C followed by 40 cycles of 20 s at 95 °C, 30 s at 60 °C, and 20 s at 72 °C. *UBI3* (*Solyc01g056840*) was used as the internal control for qRT-PCR. The primers used for qRT-PCR are listed in Supplementary Table S4.

### RNA-seq analysis

Total RNA was isolated from reproductive meristematic tissues collected from 21-d-old seedlings of ST024, *tm3cr-1*, *stm3cr-1*, and *tm3 stm3cr-1*. Three biological replicates were performed, and each biological replicate contained at least 200 individuals. A total of twelve RNA-Seq libraries were constructed and sequenced using Illumina HiSeq2000 at Berry Genomics (http://www.berrygenomics.com/). The filtered clean reads were aligned to the tomato genome (ITAG4.0) by STAR v2.5.3, and their features were counted by feature Counts v 1.5.3, as described in a previous paper^51^. The statistical package DEGseq with the MA-plot-based method in R version 3.0.3 was used to calculate the *P* value, which was adjusted using the Benjamini-Hochberg procedure. The fold change between the ST024 and *tm3cr-1*, ST024 and *stm3cr-1*, and ST024 and *stm3 tm3cr-1* libraries was calculated as FPKM (fragments per kilobase of transcript sequence per million base pairs sequenced). The thresholds for the identification of DEGs were as follows: FPKM > 1 in any tissue, fold change > 1.5 or < 0.6666, and Benjamini-Hochberg adjusted *P* value < 0.05. GO analyses of DEGs were performed using their best homologous genes in *Arabidopsis* with DAVID (Database for Annotation, Visualization, and Integrated Discovery, https://david.ncifcrf.gov/).

### RACE assay

A SMARTer RACE cDNA Amplification Kit (Clontech, 634923) was used for the RACE assay according to the manufacturer’s instructions. Approximately 1 μg of total RNA was used to prepare RACE-Ready first-strand cDNA. Then, the 3′ and 5′ ends of cDNA were amplified by using the 3′ or 5′ gene-specific primers listed in Supplementary Table S4. The obtained PCR products were ligated to vectors for sequencing.

### Constructs and plant transformation

For CRISPR/Cas9 constructs, two sgRNA binding sites per gene were selected with the CRISPR-P v2.0 tool (http://cbi.hzau.edu.cn/CRISPR2/). Primers containing sgRNAs and *Bsa*I recognition sites were used to amplify the sgRNAX_U6-26t_SlU6p_sgRNAX fragments using a pCBC_DT1T2_SlU6p vector as the template, after which the fragments were purified and cloned into pTX041 at the *Bsa*I sites^52,53^. To generate *35S*::*STM3-YFP-HA*, *35S*::*TM3-YFP-HA,* and *35S*::*STM3-GFP-FLAG* constructs, the coding sequences of *STM3* and *TM3* were fused to the N-terminus of YFP-HA or GFP-FLAG, respectively. All plasmids were validated by sequencing and then transformed into the *Agrobacterium tumefaciens* strain *AGL1*. The transgenic plants were validated by PCR and sequencing. All primers used are listed in Supplementary Table S4.

### In situ hybridization

In situ hybridization was performed as described in Scott et al. with modifications^54^. The *STM3* and *TM3* cDNA segments were amplified with the primers P9 and P7, respectively (Supplementary Table S4), and then cloned into pEAZY-T3 (TransGen, CT301-01), which contained T7 and SP6 promoter sequences. In vitro transcription was performed with T7 RNA polymerase to produce the antisense or sense probe for in situ hybridization.

Meristem tissues were dissected by hand and fixed for 24 h at 4 °C in freshly prepared 4% (w/v) paraformaldehyde buffered with phosphate-buffered saline (PBS, pH 7.2). Fixed tissues were dehydrated in a graded ethanol:histochoice (Sigma, H2779) series and impregnated with paraplast (Sigma, P3683). Dewaxed thin sections (10 μm) were hybridized with hydrolysis probes for 12 h at 55 °C. Complete color development sections were observed using a fluorescence microscope (Leica, DM5500).

### Yeast two-hybrid

Yeast two-hybrid assays were performed according to the Yeastmaker™ Yeast Transformation System 2 user manual (Clontech, PT1172-1). To explore interactions between STM3, TM3, and itself, the full-length coding sequence of each gene was cloned into bait and prey vectors, pGBKT7 and pGADT7, respectively. A pair of bait and prey plasmids were cotransformed into the Y2H gold yeast strain according to the Clontech yeast protocol handbook instructions. The resultant strains were subsequently grown on plates lacking leucine and tryptophan for 3 days at 30 °C. The interaction was tested via growth assays on media lacking leucine, tryptophan, histidine, and adenine but containing X-*alpha*-Gal.

### Co-immunoprecipitation assay

The Co-IP assay was performed as previously described with minor modifications^55^. The coding sequences of *STM3* and *TM3* were fused to the N-terminus of FLAG or YFP-HA tags to construct *35S*::*STM3-FLAG*, *35S*::*STM3-YFP-HA*, and *35S*::*TM3-YFP-HA* vectors. These plasmids were transformed into *A. tumefaciens* strain *EHA105* and then coinfiltrated into the leaves of 4-w-old *N. benthamiana* plants together with P19. Equal numbers of samples were collected two days after infiltration, ground in liquid nitrogen, and then homogenized in 1 ml of extraction buffer: 50 mM Tris-HCl, pH 7.5; 150 mM NaCl; 0.19% CA630; 20% glycerol; 5 mM DTT; and 1 tablet/50 ml of protease inhibitor cocktail (Roche, 04693132001). The lysates were mixed sufficiently and then centrifuged at 16,000 × *g* for 10 min at 4 °C. In addition, 5 μg anti-HA (Sigma, H6908) antibody and 20 μl Dynabeads Protein G (Novex, 10001D) were incubated for 2 h at 4 °C. The lysates and the HA-binding beads were incubated overnight at 4 °C. The incubated beads were washed five times with 1 × PBS. Then, 30 μl of 6× protein loading buffer was added and boiled for 5 min. The proteins were electrophoretically separated by 10% SDS–PAGE and transferred to a PVDF membrane (Immobilon-P, IPVH00010). Immunoblots were performed using an anti-FLAG antibody (MBL, M185-3L, 1:2000) for STM3-FLAG. The bands were visualized on a Tanon-5200 Chemiluminescent Imaging System (Tanon Science and Technology).

### ChIP and ChIP-qPCR

ChIP was performed using 0.5 g hand-dissected meristems of 18-d-old seedlings of *35S*::*STM3-YFP-HA* transgenic plants as described previously with minor modifications^32^. Meristems were completely ground in liquid nitrogen and cross-linked in 1% formaldehyde (Sigma-Aldrich) for 10 min at 4 °C. Chromatin was sheared using a Diagenode Bioruptor Plus instrument to obtain ~300 bp DNA fragments. Anti-HA (Sigma, H6908) was used for immunoprecipitation. The DNA isolated by ChIP was used for qPCR analysis or Illumina paired-end sequencing. qPCR was performed using EvaGreen 2×qPCR MasterMix-No Dye (Abm, MasterMix-S) on a Bio-Rad CFX-96 Real-Time PCR instrument with the following program: 3 min at 95 °C followed by 50 cycles of 20 s at 95 °C, 30 s at 60 °C, and 20 s at 72 °C. The intergenic region around *ACTIN* (*Solyc03g078400*) was used as an internal control. Primers for qPCR are listed in Supplementary Table S4.

### Electrophoretic mobility shift assay

The full-length coding regions of *STM3* were amplified by PCR using the primer pair in Supplementary Table S4. The PCR product was ligated into the MBP-pMCSG7 plasmid containing a polyhistidine (6×His) sequence by a ligation-independent cloning method as previously described^56^. All recombinant proteins were expressed and purified from *Escherichia coli* strain *BL21 RIL* (*BL21* CP, Stratagene). The MBP-STM3 protein and MBP protein were induced by 0.2 mM isopropylthio-b-D-galactoside at 16 °C for 16 h. Ni-NTA agarose (QIAGEN, 1018244) was used to purify MBP-STM3 and MBP according to the manufacturer’s instructions. We synthesized and annealed the 5′ biotin DNA probes used in EMSA. DNA gel shift assays were performed using the LightShift Chemiluminescent EMSA kit (Thermo Fisher Scientific, 20148). Each EMSA binding reaction (20 μL) contained 0.5 μL purified recombinant protein, 4 μL biotin-labeled probe DNA (100 pmol), 2 μL binding buffer, and 1 μL poly (dI-dC). Transferred DNA and protein were cross-linked using a UV lamp at 312 nm. The biotin-labeled DNA was determined using a Thermo Scientific chemiluminescence kit. The bands were visualized on a Tanon-5200 Chemiluminescent Imaging System (Tanon Science and Technology).

### Dual-luciferase reporter assay

For plasmid construction, the pluc-35Rluc backbone vector was obtained from pPZP211^57^. The nearly 2.3 kb *FUL1* promoter sequence was amplified using MM genomic DNA as a template and integrated into pluc-35Rluc using the in-fusion HD cloning kit (Clontech, 639649). Then, the mutated *FUL1* promoter was generated by deleting the 40 bp sequence including the CArG box in the S1 region of the promoter (Fig. 6b). The primers used for the constructs are listed in Supplementary Table S4. The plasmids were transformed into *Agrobacterium tumefaciens EHA105* competent cells. A single colony was cultured in Luria-Bertani (LB) medium until the OD_600_ value reached 1. The *A. tumefaciens* cells were collected by centrifugation and suspended using 10 mM MgCl_2_ and 150 μM acetosyringone. The cells containing the overexpression plasmids, luciferase plasmid, and p19 plasmid were mixed in a volume ratio of 2:1:3 and infiltrated into *Nicotiana benthamiana* leaves using a syringe. The leaves were harvested and ground in liquid nitrogen at 2 d after infiltration. The activities of firefly luciferase and *Renilla* luciferase were measured using a dual-luciferase reporter assay system (Promega; E1910) on a Promega GLOMAX 20/20 LUMINOMETER. Thirty-seven sets of data were collected from the different leaves. The measured data (LUC/REN) were normalized to the control on the same leaf.

## Supplementary information

supplementary table S1

all supplementary figures

Supplementary Table S2

Supplementary Table S3

Supplementary Table S4

## Data Availability

The RNA sequencing datasets generated in this study have been deposited in the Sequence Read Archive (SRA) under the accession number PRJNA706044. Other data supporting our findings are available in the manuscript file or from the corresponding author upon request.
