# Supplementary material for: SISTER OF TM3 activates FRUITFULL1 to regulate inflorescence branching in tomato
Source: Hortic Res. 2021 Dec 1;8:251. doi: 10.1038/s41438-021-00677-x (PMC8633288; doi:10.1038/s41438-021-00677-x)
Supplement: Supplementary file 1 — supplementary table S1 [file 41438_2021_677_MOESM1_ESM.docx]

Supplementary Table S1. Statistical results of the RNA-seq data

| **Sample name** | **Total reads** | **Uniquely mapped reads** | **Uniquely mapped reads %** |
| --- | --- | --- | --- |
| ST024-1 | 32603145 | 31012556 | 95.12% |
| ST024-2 | 33414043 | 31733078 | 94.97% |
| ST024-3 | 35686433 | 33984309 | 95.23% |
| *stm3cr-1* | 35028611 | 33466369 | 95.54% |
| *stm3cr* -2 | 35074692 | 33499526 | 95.51% |
| *stm3cr* -3 | 33039034 | 31068175 | 94.03% |
| *stm3 tm3cr* -1 | 32444050 | 31028233 | 95.64% |
| *stm3 tm3cr* -2 | 30878721 | 29149443 | 94.40% |
| *stm3 tm3cr* -3 | 33374729 | 31583649 | 94.63% |
| *tm3cr*-1 | 30979089 | 29612839 | 95.59% |
| *tm3cr*-2 | 32285237 | 30881240 | 95.65% |
| *tm3cr*-3 | 32179973 | 30819526 | 95.77% |
