# Supplementary material for: SISTER OF TM3 activates FRUITFULL1 to regulate inflorescence branching in tomato
Source: Hortic Res. 2021 Dec 1;8:251. doi: 10.1038/s41438-021-00677-x (PMC8633288; doi:10.1038/s41438-021-00677-x)

Supplementary Fig.S1

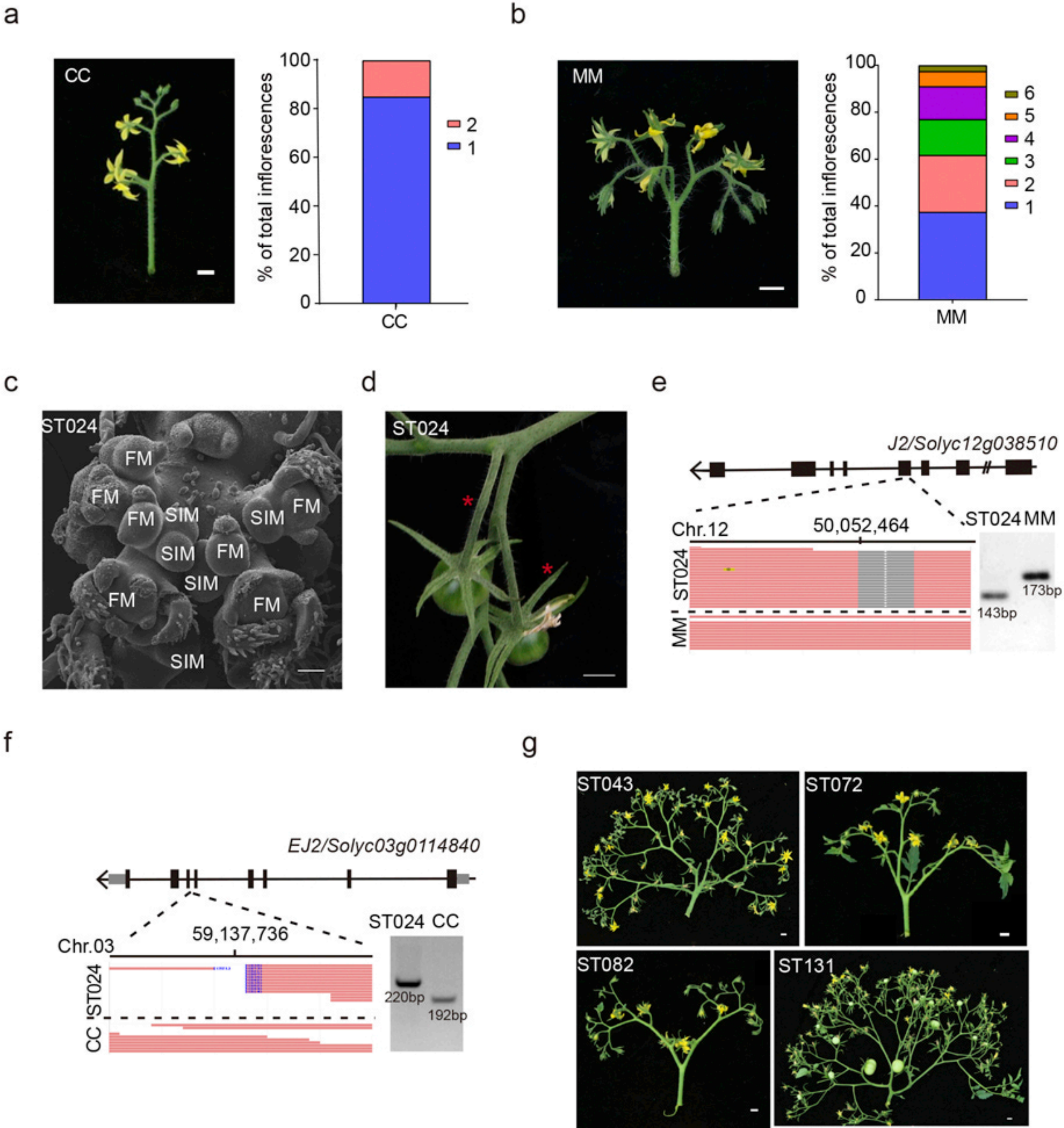

Supplementary Fig.S2

a

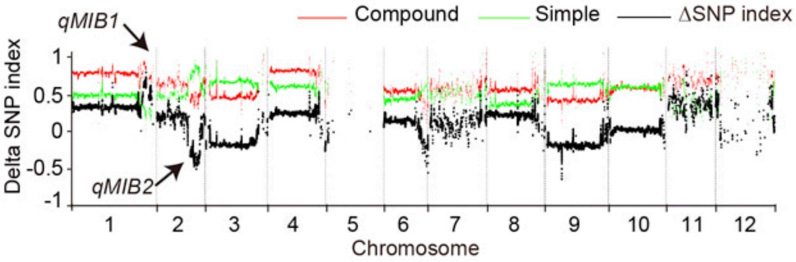

b

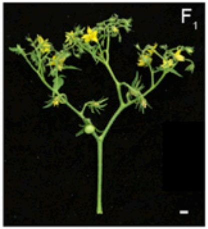

c

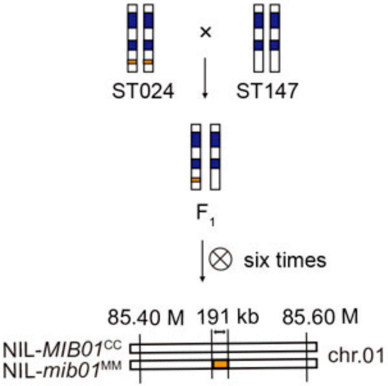

Supplementary Fig.S3

a

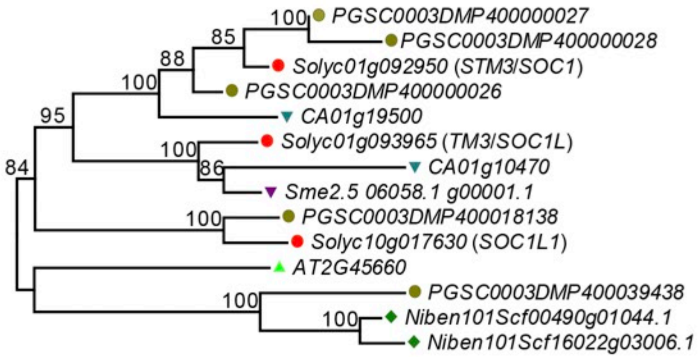

b

STM3 cDNA sequence

GTCTGAGATTTTTCTATATTTGGGATTGATCTAAAGATATGGTTCGAGGTAAA
ACCCAGATGAGGCGTATAGAGAACGCCACAAGCAGACAAGTTACTTTCTCAAA
GAGAAGAAATGGTTTGCTAAAAAAAGCTTTTGAGCTTTCTGTCTTATGTGATG
CTGAAGTGGGATTGATTATATTTTCTCCAAGAGGAAAACTATATGAATTTGCT
AGTTCAAGCATACCTGAGGTAATTGAACGATATAAGAGGCATACTAAAGATAA
AGTTAAATCTGATGAAAATCAATCTGTGGATATTCAGCATACTAAGCAAGAGA
CAGCAAGTTTGATGAAGAAGATAGAACTCCTTGAATCATCTAAAAGGAAACTC
TTGGGAGAAGGTTTAGGATCATGCAGCCTTGAAGAAGTTCAACAATTAGAGAA
GCAATTGGAGCAGAGTGTCATTACTATTAGGGCTCGAAAGATGCAAGTCTTTA
GGGAACAAATGGAAAGATTGAAAGAAAGGGAGAGAGCCCTTACAGCTGAAAAT
ATGATGCTGAGGGAACTAAAGTTTGGAGGAGATGAAGAGAGACGAAAATCAAG
TGGAGAGAAAGAAAGAGAAGTAGTTTTTTGTATAGAAGGTGGTAGTACTGGTA
GCGAAAAATCAGATGTTGAGACTGAATTGTTTATCGGACAACCTCATTATCAT
GACTCCAGAATAAGGCGTCCTGAATGGTCTTGAGTGGTTCGATATAGGAACCA
CTCAAGACCGAAGAATTGCTCTCAAGTACCCTTATTTTGTACTTTGTATAAAT
TTTGACTAATTATCAAGTATAAACTAACTAGTAATCTCAGCTGCTAAATTATT
TTGGAGATGTATGAGTGGGTTTAATTTCTCTCTTTAATTAATTAAGATGG

c

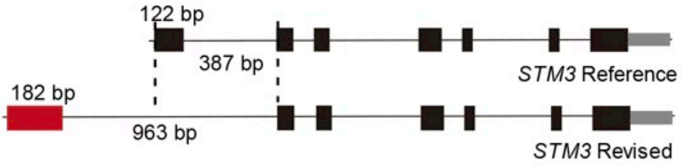

Supplementary Fig.S4

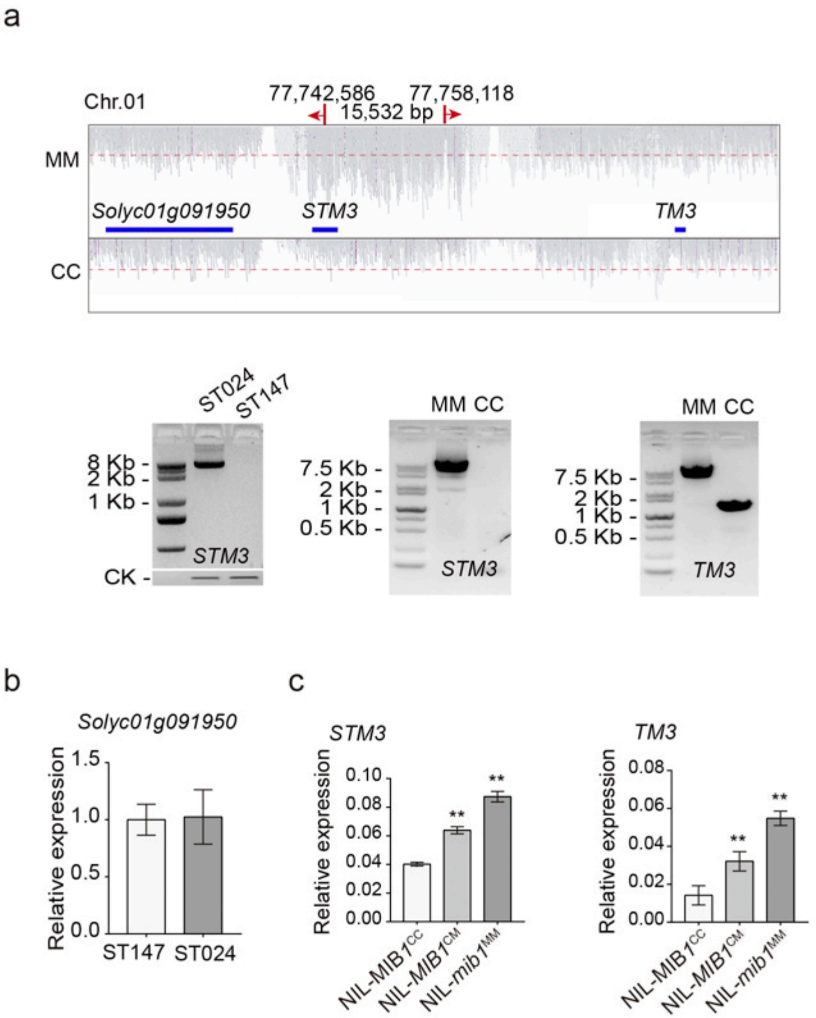

Supplementary Fig.S5

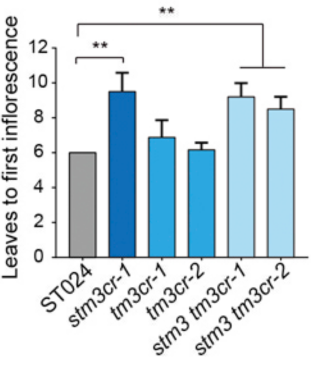

Supplementary Fig.S6

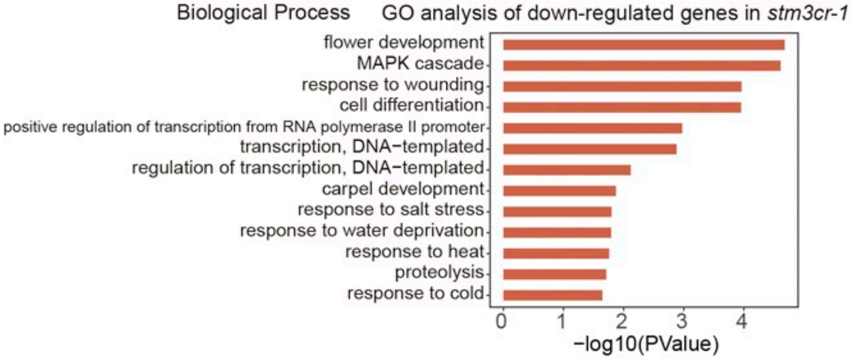

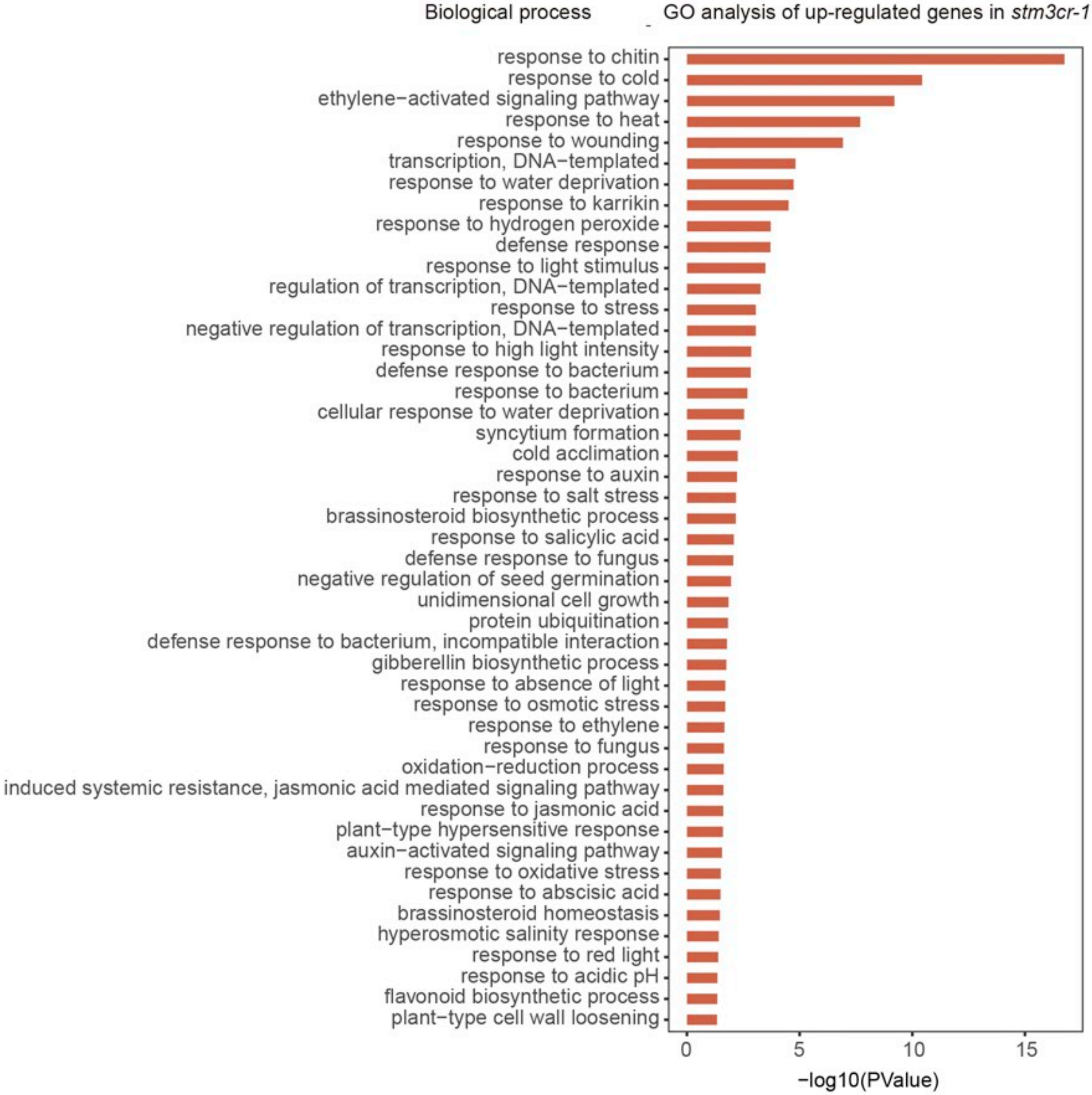

Supplementary Fig.S7

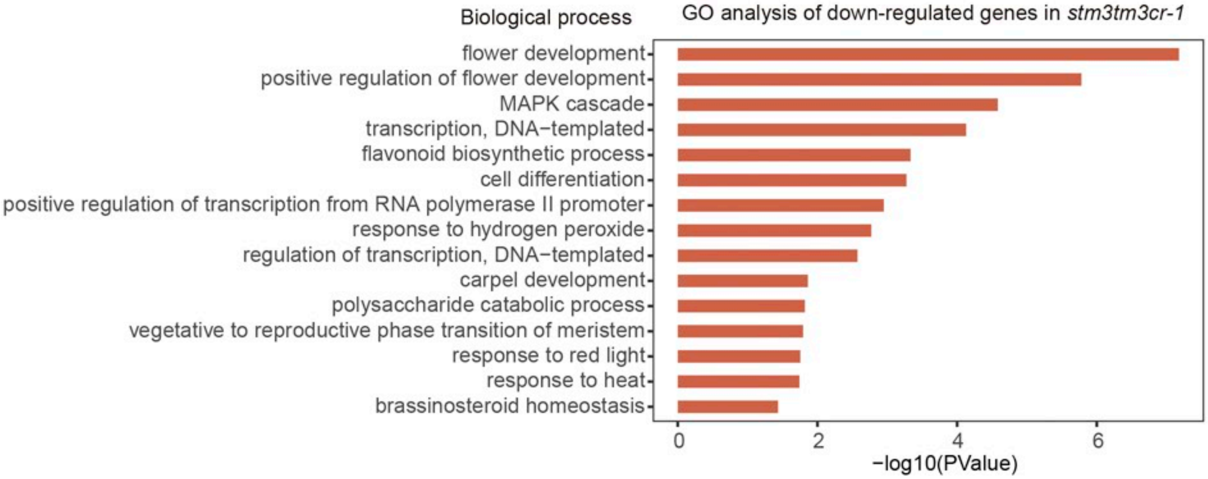

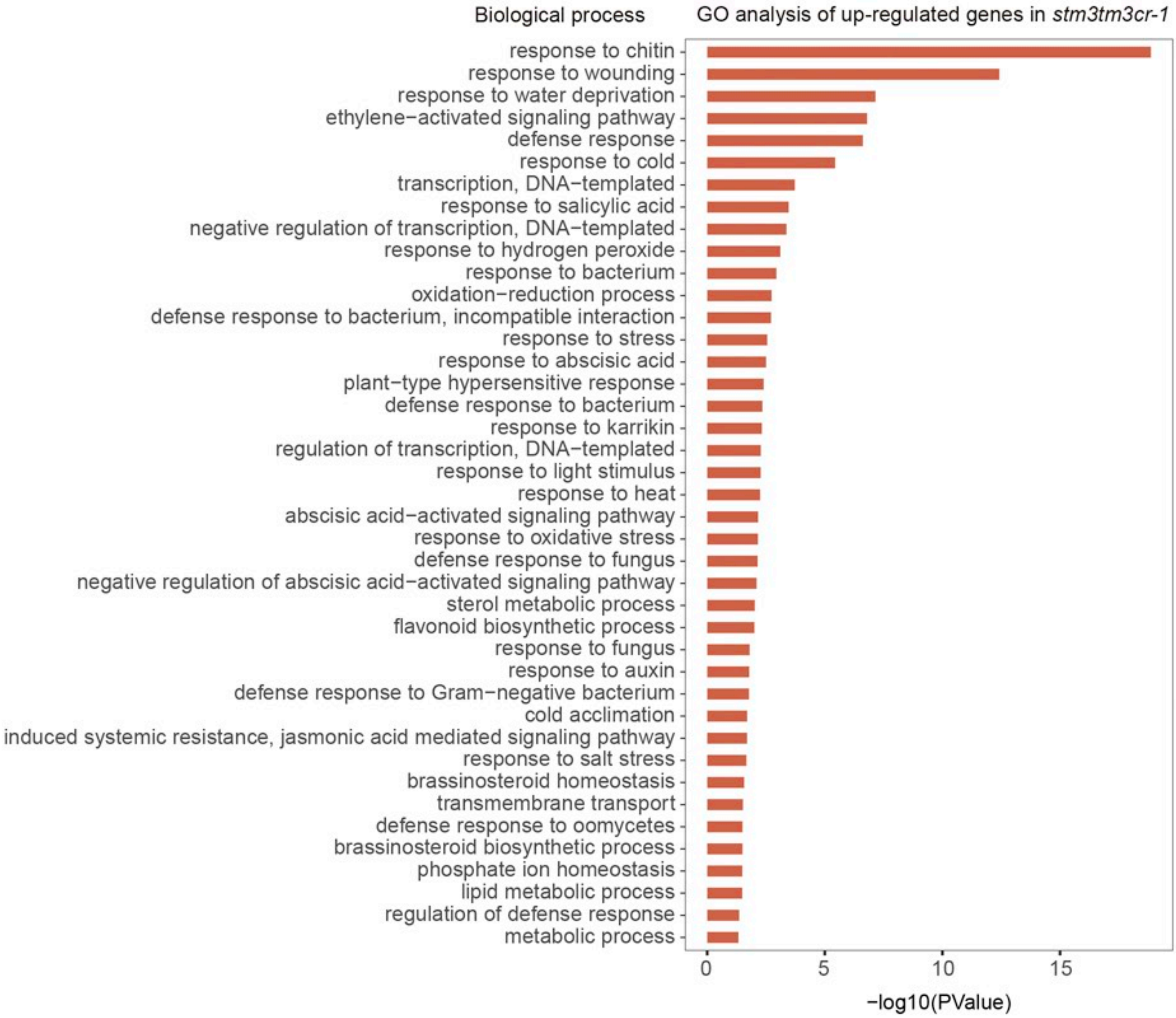

Supplementary Fig.S8

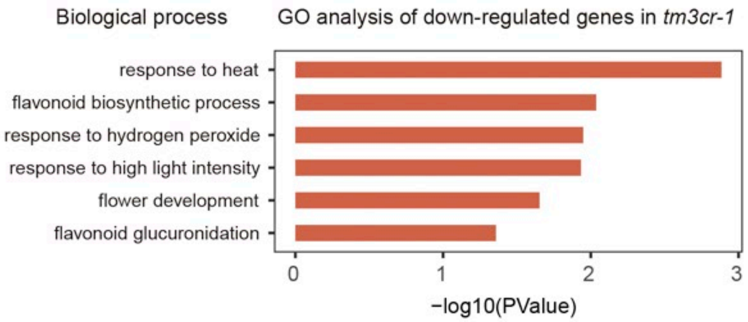

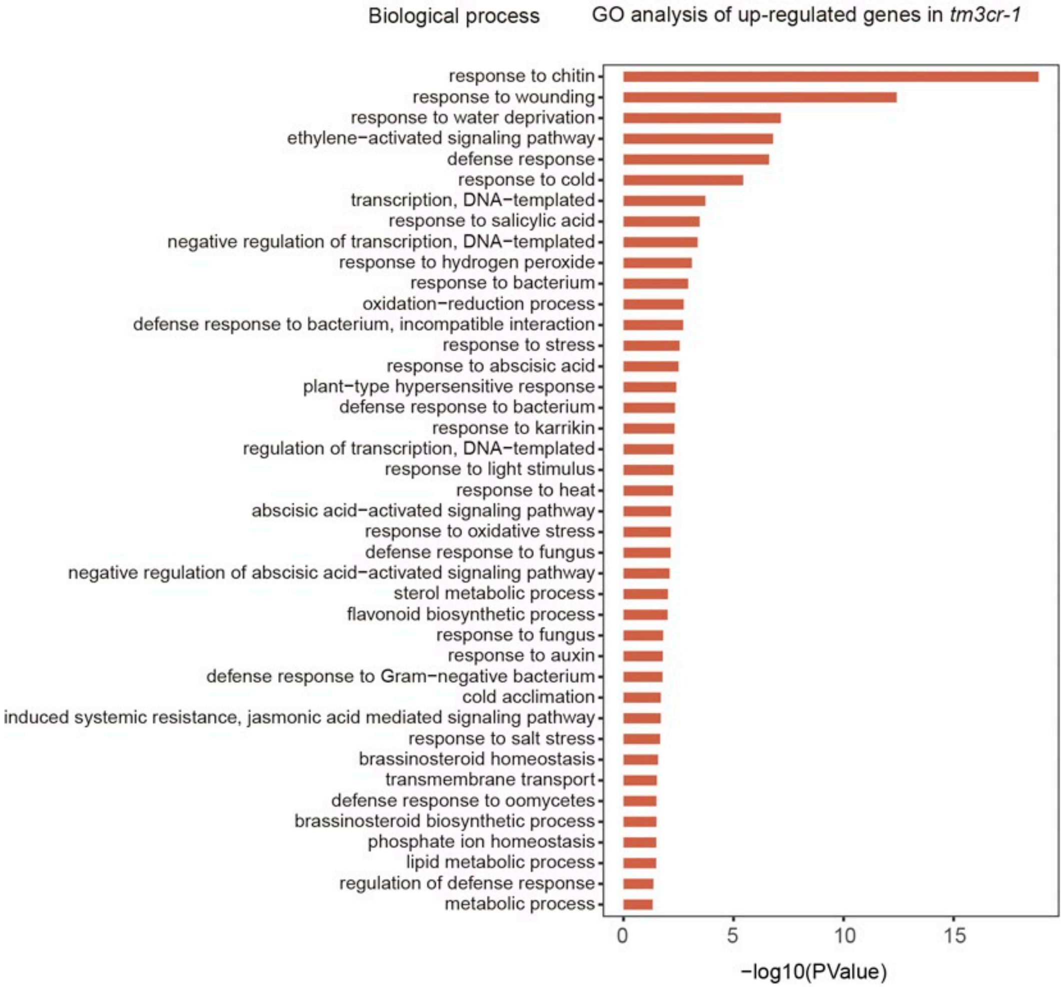

Supplement: Supplementary file 2 — all supplementary figures [file 41438_2021_677_MOESM2_ESM.pdf]
